# Trends in the surgical treatment of proximal humeral fractures – a nationwide 23-year study in Finland

**DOI:** 10.1186/1471-2474-13-261

**Published:** 2012-12-29

**Authors:** Tuomas T Huttunen, Antti P Launonen, Harri Pihlajamäki, Pekka Kannus, Ville M Mattila

**Affiliations:** 1 Department of Anesthesia, Valkeakoski Regional Hospital, Valkeakoski, Finland; 2 Division of Orthopaedics and Traumatology, Department of Trauma, Musculoskeletal Surgery and Rehabilitation, Tampere University Hospital, Tampere, Finland; 3 Division of Orthopaedics and Traumatology, Seinäjoki Central Hospital, Seinäjoki, Finland; 4 Injury & Osteoporosis Research Center, UKK Institute for Health Promotion Research, Tampere, Finland; 5 Medical School, University of Tampere, Tampere, Finland

## Abstract

**Background:**

Proximal humeral fractures are common osteoporotic fractures. Most proximal humeral fractures are treated non-surgically, although surgical treatment has gained popularity. The purpose of this study was to determine changes in the surgical treatment of proximal humeral fractures in Finland between 1987 and 2009.

**Methods:**

The study covered the entire adult (>19 y) population in Finland over the 23-year period from 1st of January 1987 to 31st of December 2009. We assessed the number and incidence of surgically treated proximal humeral fractures in each year of observation and recorded the type of surgery used. The cohort study was based on data from Finnish National Hospital Discharge Register.

**Results:**

During the 23-year study period, a total of 10,560 surgical operations for proximal humeral fractures were performed in Finland. The overall incidence of these operations nearly quadrupled between 1987 and 2009. After the year 2002, the number of patients treated with plating increased.

**Conclusion:**

An increase in the incidence of the surgical treatment of proximal humeral fractures was seen in Finland in 1987–2009. Fracture plating became increasingly popular since 2002. As optimal indications for each surgical treatment modality in the treatment of proximal humeral fractures are not known, critical evaluation of each individual treatment method is needed.

## Background

Proximal humeral fractures are common and they are the third most common osteoporotic fracture after hip and distal radius fractures [1-3]. The rate of proximal humeral fractures typically increases in women after age 50 and in men after age 70 [4]. Based on recent literature, the age- and sex-specific incidence rate of proximal humeral fractures varies from 10 to 300 per 100,000 person-years in different populations [1,2,4,5].

Proximal humeral fractures typically occur due to a low-energy trauma, most commonly by falling from standing height [6]. The incidence of proximal humeral fractures has clearly increased over the past few decades [5,7]. Despite the high prevalence of these injuries, surprisingly little is known which proximal humeral fractures should be treated surgically [8].

Most proximal humeral fractures are treated nonsurgically [1,9,10]. A variety of different methods can be used for surgical treatment of proximal humeral fractures, including percutaneous fixation, open reduction and internal fixation (ORIF), and arthroplasty. While there are a few clinical case series of surgical treatment few high-quality randomized controlled trials have been performed [11].

Fjalestad and coworkers found no evidence of a difference between surgical and conservative treatment, whereas Olerud and coworkers reported that arthroplasty is associated with a better quality of life. In another study Olerud et al. compared plating to conservative treatment but found no statistical difference for quality of life in elderly patients [11-14].

New treatment options, such as locking plates, were introduced to clinical practice during the recent decade, but their superiority over other treatment options has not yet been demonstrated [8,11].

The aim of the current study was to assess the incidence and trends in the surgical treatment of fractures of the proximal humerus. We were especially interested to see how the number and incidence of different surgical treatment methods have evolved at this site.

## Methods

The Institutional Review Board approved the study.

Patient data were obtained from the Finnish National Hospital Discharge Register (NHDR) between 1987 and 2009. All patients 20 years of age or older admitted to hospitals alive were included. The Finnish NHDR, founded in 1967, provides data on age, sex, domicile of the subject, hospital stay duration, primary and secondary diagnosis, and operations performed during the hospital stay. The data collected by the NHDR is mandatory for all hospitals, including private, public, and other institutions. The validity of the NHDR is excellent regarding both coverage and accuracy of the database [15-17]. On the other hand, the NHDR is a *hospital discharge register* and it does not provide conclusive data on co-morbities and other risk factors for fractures.

Patients were selected in the study if they had either primary or secondary diagnosis of a proximal humeral fracture. As the ICD-coding changed during the study period, ICD-9 codes 81200 and 81210 were used to select patients in the study between 1987 and 1995. ICD-10 code S42.2 was used to select patients in the study between 1996 and 2009. The main outcome variable for the study was the number of patients undergoing surgical treatment of a proximal humeral fracture. The procedural codes also changed during the study period. The ICD-9 was used in Finland from 1987 to 1997. During this period, we included ICD-9 surgical treatment codes 9126 (closed reduction and osteosynthesis), 9128 (open reduction and osteosynthesis), 9130 (external fixation), and 9132 (endoprosthesis). In 1998, the more specific ICD-10 procedural coding system was introduced. The ICD-10 surgical treatment codes for the proximal humeral fractures included NBJ60 (open reduction and osteosynthesis by nailing), NBJ62 (open reduction and plating), NBJ64 (fracture reduction and screw, percutaneous pinning or absorbable screw fixation), NBJ70 (external fixation), and NBB10-20 (arthroplasty). For analysis of the data for the whole study period from 1987 to 2009, the codes of the ICD-9 system were pooled with those of the ICD-10 system, and surgical treatment was categorized into four groups; closed reduction and osteosynthesis (codes 9126 and NBJ64), open reduction and osteosynthesis (codes 9128, NBJ60, and NBJ62), fracture reduction and external fixation (codes 9130 and NBJ70), and arthroplasty (codes 9132 and NBB10-20).

Implementation of the ICD-10 in 1998 allowed us to further dissect the proximal humeral procedures, and therefore a more specific analysis was performed for the years 1998 to 2009 to specify the proportions of individual surgical procedures. For this period, from 1998 to 2009, the numbers and incidences of procedures NBJ60, NBJ62, NBJ64, NBJ70, and NBB10-20 were analysed individually.

### Statistical analysis

To compute the incidence ratios of proximal humerus fractures requiring surgical intervention and thus leading to hospitalization, the annual mid-population was obtained from the Official Statistics of Finland, an electronic national population register [18]. The rates of surgically treated proximal humerus fractures (per 100,000 persons) were based on the entire adult population of Finland rather than cohort-based estimates and thus 95% confidence intervals were not calculated. Statistical analysis was performed using PASW19.0®.

## Results

A total of 47,960 hospitalizations with a diagnosis of proximal humeral fracture were registered in the NHDR during the 23-year study period. The number of patients was 1136 in 1987 and 2944 in 2009. The incidence of hospitalization following proximal humeral fracture increased from 31.1 per 100,000 person years in 1987 to 71.5 per 100,000 person years in 2009.

**Figure 1:**
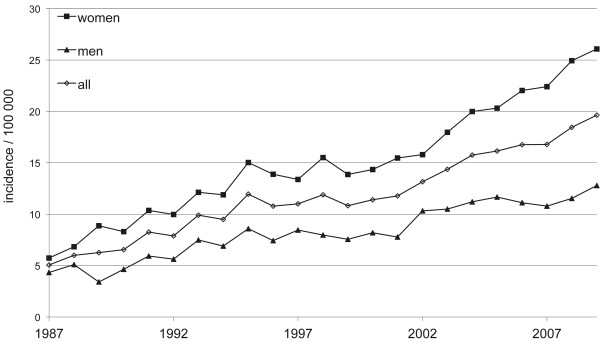
Incidence of surgically treated proximal humeral fractures in Finnish men and women per 100,000 person-years between 1987 and 2009.

During the 23-year period, 10,560 surgical operations of these fractures were registered in the NHDR. The number of surgically treated proximal humerus fractures increased from 1987 to 2009. The number of surgical procedures in women was roughly twice that in men (n = 7008; 66% in women and n = 3552; 34% in men). The total incidence of surgical procedures was 5.1 per 100,000 person years (n = 185) in 1987 and 19.6 per 100,000 person years (n = 808) in 2009. In women, the incidence increased from 5.7 per 100,000 person years (n = 110) in 1987 to 26.1 per 100,000 person years (n = 553) in 2009. In men, the incidence increased from 4.3 per 100,000 person years (n = 75) in 1987 to 12.8 per 100,000 person years (n = 255) in 2009 (Figure 1).

During the entire 23-year study period, ORIF was the most common surgical procedure performed (n = 7774, 73.6%), followed by closed reduction and osteosynthesis (n = 1515, 14.3%), arthroplasty (n = 1198, 11.3%), and external fixation (n = 73, 0.7%). As the number and incidence of external fixations were so low during the entire study period, they were excluded from further analysis.

**Figure 2:**
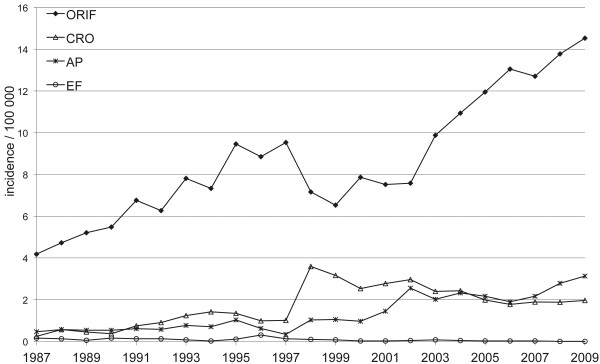
**Changes in the incidence of surgically treated proximal humeral fractures in Finnish adults per 100,000 person-years from 1987 to 2009.** ORIF = open reduction and internal fixation, CRO = closed reduction and osteosynthesis, AP = arthroplasty, EF = external fixation.

The number and incidence of different surgical procedures changed markedly (Figure 2). The incidence for ORIF was 4.2 per 100,000 person years (n = 153) in 1987 and 14.5 per 100,000 person years (n = 598) in 2009. The steepest rise in the number and incidence of the ORIF was observed among women: from 4.4 per 100,000 person years (n = 84) in 1987 to 19.1 per 100,000 person years (n = 405) in 2009. The incidence of closed reduction and osteosynthesis was 0.25 per 100,000 person years (n = 9) in 1987 and 2.0 per 100,000 person years (n = 81) in 2009. The corresponding values for arthroplasty were 0.5 (n = 17) and 3.1 (n = 129).

**Figure 3:**
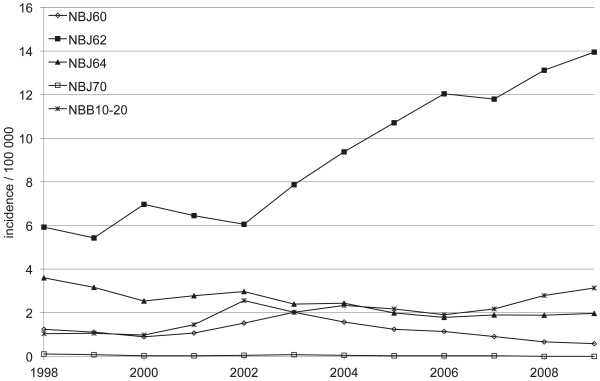
**Incidence of plating for proximal humeral fractures in Finnish adults per 100,000 person-years between 1998 and 2009.** NBJ60 = nail, NBJ62 = plate, NBJ64 = screw, NBJ70 = external fixation, NBB10-20 = arthroplasty.

**Figure 4:**
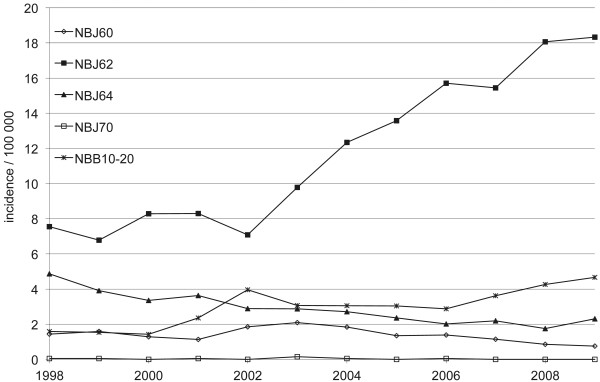
**Incidence of surgically treated proximal humerus fractures in Finnish female adults per 100,000 person-years between 1998 and 2009.** NBJ60 = nail, NBJ62 = plate, NBJ64 = screw, NBJ70 = external fixation, NBB10-20 = arthroplasty.

**Figure 5:**
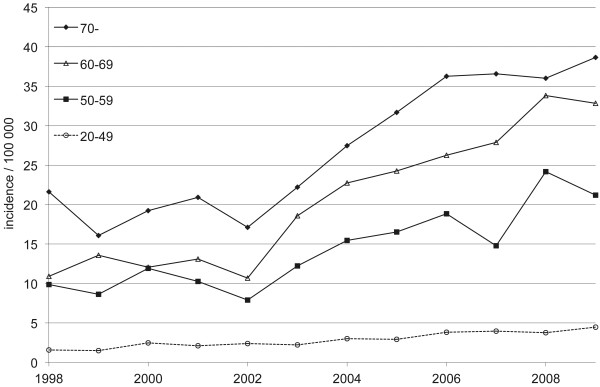
Age-specific incidence of platings for proximal humeral fractures in Finnish female adults per 100,000 person-years between 1998 and 2009.

Between 1998 and 2009, when the more specific ICD-10 codes were available, the incidence in plating (NBJ62) increased from 5.9 per 100,000 person years (n = 229) in 1998 to 13.9 per 100,000 person years (n = 574) in 2009 (Figure 3). The increase in plating was greater in women as the incidence rose from 7.6 per 100,000 person years (n = 152) in 1998 to 18.3 per 100,000 person years (n = 389) in 2009 (Figure 4). The plating incidence nearly doubled in every age group between 1998 and 2009 (Figure 5).

The incidence of nailing (NBJ60) decreased over time, from 1.2 per 100,000 person years (n = 48) in 1998 to 0.6 per 100,000 person years (n = 24) in 2009 (Figure 3). The corresponding values for fracture reduction with screw, and percutaneous pinning or absorbable screw fixation (NBJ64) were 3.6 (n = 139) and 2.0 (n = 81). The incidence of arthroplasty (NBB10-20) increased from 1.0 (n = 40) in 1998 to 3.1 per 100,000 person-years (n = 129) in 2009 (Figure 3). The mean age by surgery type varied: 65.0 yrs. (SD 15) for nailing, 61.7 yrs. (SD 15) for plating, 59.3 yrs. (SD 16) for screw, pin or absorbable screw, 68.1 (SD 12) for external fixation and 69.5 (SD 11) for arthroplasty.

## Discussion

In this cohort study based on a nationwide register, we analysed the trends for surgical treatment of proximal humeral fractures in the entire adult Finnish population. The main finding was that the incidence of surgical treatment of proximal humeral fractures nearly quadrupled between 1987 and 2009. This is of interest as proximal humeral fracture is the third most common osteoporotic fracture type and as such poses considerable strain on our healthcare system. At the same time the incidence of hospitalization due to proximal humeral fractures only doubled, and more specifically, in the oldest age groups the age-adjusted incidence of these fractures has stayed quite constant since the late 1990s [5].

A majority of proximal humeral fractures occur in women with incidence increasing almost exponentially with aging [19,20]. According to our study the incidence for surgical treatment rose for both men and women but it is unclear why the rise in incidence is steeper with women. Aging women have shown to have a greater risk than men for an osteoporotic fracture such as proximal humeral fractures [21,22].

Surprisingly little is known regarding whether two, three, or four part humeral fractures in elderly patients should be treated operatively or conservatively [8,11]. There are few randomized controlled trials comparing nonsurgical versus surgical treatment with adequate scoring in follow-up reports [12-14]. In light of the scarce evidence, the significant increase in plating that occurred after the introduction of locking plates in Finland in 2002 is noteworthy. The number and incidences of ORIF with plating more than doubled between 1998 and 2009. These findings may imply that orthopaedic surgeons adopt new fixation systems without conclusive evidence or knowledge whether these fractures should be treated surgically at all. In a previous independent study we observed a significant increase in the surgical treatment of humeral shaft fractures [23]. The change in the rate of surgical treatment was not as drastic as in the current study on proximal humeral fractures.

The small number of arthroplasty in the surgical treatment of proximal humeral fractures was surprising as based on the literature, joint replacement is usually suggested especially in age groups of 70 years and older [24]. The incidence of arthroplasty was quite steady from the late 80’s until the late 90’s. The incidence has since risen (Figure 4) but not as sharply as plating. At the same time fracture plating in women over 70 has gained popularity (Figure 5).

In Finland, medical treatment is equally available to everyone and the study population comprised the entire Finnish adult population; therefore, we consider our study reliable. In addition, previous studies reported the coverage and accuracy of the NHDR injury codes to be over 90% [17]. A strength of our study is the excellent national coverage of surgically treated proximal humeral fractures; all surgically treated proximal humeral fractures between 1987 and 2009 are included in this study, whether treated as outpatients or inpatients.

A weakness of this study is that the precise incidence of all proximal humeral fractures cannot be assessed using the NHDR data alone because an unknown number of the fractures were treated conservatively on an outpatient basis. Thus we are not able to deduct whether a part of the increase in the incidence of operative treatment of proximal humeral fractures is due to growing numbers of proximal humeral fractures or a growing tendency towards surgical treatment. The available scientific literature suggests that the majority of proximal humeral fractures are still treated nonsurgically [10,25]. Another limitation of our 23-year study is the change in the ICD procedure-coding system in 1998. Due to the less specific procedural codes in the ICD-9 system, specific data about the implants (i.e., pinning, plates) used could not be evaluated during 1987–1997. Because of this, the main finding of this study between 1987 and 1997 is the increase in the incidence of surgical treatment of proximal humeral fractures. The implementation of locking plates in Finland occurred at the beginning of the 2000s when the more specific ICD-10 coding system was already in use.

In Finland the use of procedural coding of humeral fracture surgery is exercised as explained in Methods but the practical use of procedural coding between different countries may vary. For instance plating of humeral fracture in Finland is NBJ62 but NBJ61 in Norway. The possible differences in procedural coding have to therefore be taken into account when comparing results between different countries.

According to Bell and co-workers, the incidence of surgical treatment for proximal humeral fractures has increased in North America [10]. With the lack of consensus on the treatment of choice for proximal humeral fractures, this increased incidence of surgical treatment seems controversial, especially for the older age groups. The lack of evidence makes it difficult to determine whether ORIF with plating is the best surgical treatment option. According to our data, with the exception of plating and arthroplasty, the incidence of all other surgical treatment options has decreased with time, consistent with the findings of Bell et al. [10].

## Conclusions

Given the scarce amount of evidence concerning surgical versus nonsurgical treatment of proximal humeral fractures, the marked increase in plating procedures performed after the introduction of locking plates in 2002 is noteworthy. In clinical practice good functional outcome and patient satisfaction in shoulder-specific questionnaires, and minimal rate of complications and reoperations should be characteristic for surgical treatment of the proximal humeral fractures. To assess whether (or which) surgical treatment provides this we need more high-quality prospective randomised clinical studies with adequate follow-up.

## Competing interests

The authors declare no competing interests.

## Authors' contributions

TH and VM were in charge and contributed in all stages of the study. AL contributed in data interpretation and writing the final manuscript. PK and HP contributed in study design, data collection, data interpretation and writing the final manuscript. All authors read and approved the final manuscript.

## Source of funding

No external funding was received for or in the course of this study.

## Pre-publication history

The pre-publication history for this paper can be accessed here:

http://www.biomedcentral.com/1471-2474/13/261/prepub
